# Performance of Diagnostic Biomarkers for Traumatic Brain Injury Within the First Hour—Expanding Their Clinical Utility

**DOI:** 10.1001/jamanetworkopen.2024.31102

**Published:** 2024-09-03

**Authors:** Ramon Diaz-Arrastia

**Affiliations:** Department of Neurology, University of Pennsylvania Perelman School of Medicine, Philadelphia, Pennsylvania

The use of biomarkers measurable in blood has long been a holy grail in the clinical neurosciences. The first milestone was achieved in February 2018, when the US Food and Drug Administration (FDA) cleared the first blood test for detecting glial fibrillary acidic protein (GFAP) and ubiquitin C-terminal hydrolase L1 (UCH-L1) for use in the evaluation of mild traumatic brain injury (mTBI) to identify patients unlikely to have intracranial lesions on cranial tomography (CT) scanning, for whom CT scans are unnecessary.^1^ This assay required a 3-hour turnaround, which was not clinically practical and was thus never marketed. More recently, 4 other assays for GFAP and UCH-L1 that provide results in clinically useful times of 15 to 20 minutes have obtained regulatory clearance from the FDA and the European Union. The context of use for each of these assays is similar: as an aid to identify patients with suspected mTBI likely to have intracranial lesions on CT examination.^2^ The availability of these tests has generated much excitement in the field, and a recent National Institute of Neurological Disorders and Stroke workshop recommends incorporating biomarker tests into the classification schema for TBI.

However, implementation of these biomarker tests into clinical practice has been slow, in part because the currently cleared context of use is narrow. In well-resourced emergency departments (EDs) in developed countries, avoiding a head CT is not a highly compelling reason to implement a new blood test, since modern CTs are safe, fast, and have a long history of efficacy for identifying the few patients with mTBI who require inpatient care and neurosurgical interventions. These biomarkers will likely be useful in underresourced environments, such as far-forward military settings, remote rural areas, and lower- and middle-income countries, where CT scans are not widely available.

In this study, Papa and colleagues^3^ present data that suggest another potential context of use, namely the use of these biomarkers in prehospital settings. Assaying blood biomarkers at the scene of the injury or during ambulance transport would help inform the decision of whether the patient can safely be transported to the nearest ED, or whether the nearest ED should be bypassed because the patient is likely to need neurosurgical care that may only be accessible at a higher-level trauma center. To date, very little research has addressed this important context of use, since most studies have used blood samples collected several hours after injury. The ALERT study,^1^ for example, had a median (IQR) time for blood collection of 3.2 (2.3-4.0) hours after injury, while the time from injury to blood collection was 5.0 (3.5-9.5) hours in the ED stratum of the CENTER-TBI study^4^ and 15.6 (9.2-20.5) hours in the TRACK-TBI study.^5^ Papa et al^3^ took advantage of blood samples collected from participants in the multicenter Prehospital Tranexamic Acid Use for TBI randomized clinical trial, which was conducted under exemption from informed consent guidelines, allowing collection of blood samples for research immediately on arrival to the ED. The Prehospital Tranexamic Acid Use for TBI study enrolled only participants with Glasgow Coma Scale (GCS) score in the field of 3 to 12, thus representing a very different population than those studied in the pivotal trials that led to FDA and Conformité Européenne–mark clearance for GFAP and UCH-L1 assays. Among 804 patients included in the study by Papa et al,^3^ 375 had blood samples collected within 30 minutes, and an additional 188 had blood samples collected between 31 and 60 minutes after injury, representing the largest cohort of very early postinjury samples, to my knowledge.

The main finding of the study by Papa et al^3^ was that in these very early samples, GFAP outperformed UCH-L1 (as well as microtubule associated protein-2, which was also assayed) in identifying participants likely to have CT lesions (area under the receiver operating characteristic curve [AUC] for samples taken <30 minutes, 0.88; 95% CI, 0.85-0.92), those who needed a neurosurgical intervention within 24 hours (AUC for ≤30-minute samples, 0.78; 95% CI, 0.72-0.84), and those with clinically important early outcomes (defined as death or need for neurosurgical intervention within 7 days or need for mechanical ventilation for >7 days; AUROC for ≤30-minute samples, 0.89; 95% CI, 0.85-0.93). Combinations of the 3 biomarkers did not improve on GFAP alone. Using the suggested GFAP cutoff level for detecting CT lesions of 30 pg/mL, sensitivities of more than 98% were achieved for estimating need for neurosurgical intervention within 24 hours or clinically important outcomes by 7 days, with reasonable, clinically useful specificities. These results suggest that GFAP measurements could be useful for informing triage decisions in prehospital settings.

This important study by Papa et al^3^ raises several key questions. The first is whether UCH-L1 measurements add value, or whether GFAP alone is sufficient.^6^ While the published literature^1,4,5^ is consistent with the findings of this study by Papa et al^3^ and does not indicate that UCH-L1 levels add discriminant power over GFAP alone, most prior studies have few samples collected within the first hour of injury. Since UCH-L1 peaks earlier than GFAP,^7^ it has remained an open question of whether UCH-L1 levels are needed in the very early time period after injury. While the study by Papa et al^3^ argues against that view, the question remains open, since all the participants in the Prehospital Tranexamic Acid Use for TBI trial had GCS scores less than 13, and it remains a possibility that in mTBI, UCH-L1 adds discriminant value at very early time periods. A second question is whether other biomarkers could improve on the specificity, particularly for the need for neurosurgical interventions and clinically meaningful outcomes (mortality, need for delayed neurosurgical intervention, or prolonged mechanical ventilation), for which GFAP alone at a standard cutoff has marginal specificity of around 20%. For example, neurofilament light chain, a biomarker of traumatic axonal injury,^8^ provides prognostic information that is orthogonal to GFAP and UCH-L1^9^ and in combination with GFAP could result in improved discriminant ability. Such questions will have to wait future research focused in the prehospital space.

While there is well-justified excitement in the TBI field regarding the use of blood biomarkers in the management of TBI, the currently FDA-cleared context of use is narrow, and further research is needed on the use of blood biomarkers in others clinical scenarios, such as prehospital settings, in neurological intensive care units,^10^ and outpatient clinics. The study by Papa et al^3^ adds important new information regarding the potential for biomarkers measured in blood for prehospital and ultra-early decision-making in brain injury medicine.
